# Spontaneous Aberrant Bodies Formation in Human Pneumocytes Infected with *Estrella lausannensis*

**DOI:** 10.3390/microorganisms11102368

**Published:** 2023-09-22

**Authors:** Aurelien Rovero, Carole Kebbi-Beghdadi, Gilbert Greub

**Affiliations:** 1 Institute of Microbiology, Lausanne University Hospital and University of Lausanne, 1011 Lausanne, Switzerland; aurelienrovero@gmail.com (A.R.); carole.kebbi-beghdadi@chuv.ch (C.K.-B.); 2 Infectious Diseases Service, Department of Medicine, Lausanne University Hospital and University of Lausanne, 1011 Lausanne, Switzerland

**Keywords:** *Chlamydia*-related bacterium, replication, persistence, chronic infection, cytopathic effect

## Abstract

*Estrella lausannensis*, a *Chlamydia*-related bacterium isolated from a Spanish river, is considered as a possible emerging human pathogen. Indeed, it was recently demonstrated to multiply in human macrophages, resisting oxidative burst and causing a strong cytopathic effect. In addition, a preliminary study highlighted a correlation between antibody response to *E. lausannensis* and pneumonia in children. To clarify the pathogenic potential of these bacteria, we infected a human pneumocyte cell line with *E. lausannensis* and assessed its replication and cytopathic effect using quantitative real-time PCR and immunofluorescence, as well as confocal and electron microscopy. Our results demonstrated that *E. lausannensis* enters and replicates rapidly in human pneumocytes, and that it causes a prompt lysis of the host cells. Furthermore, we reported the spontaneous formation of aberrant bodies, a form associated with persistence in *Chlamydiae*, suggesting that *E. lausannensis* infection could cause chronic disorders in humans.

## 1. Introduction

The *Chlamydiae* phylum encompasses several families of obligate intracellular Gram-negative bacteria with varied ecological niches [1,2]. They have been isolated from a wide variety of hosts, ranging from humans to arthropods or protozoa. [3,4,5,6]. The phylum is divided into bacteria belonging to the *Chlamydiaceae* family, which includes well-known human and animal pathogens such as *C. trachomatis, C. pneumoniae* or *C. psittaci*, and *Chlamydia*-related bacteria, which encompass emerging pathogens as well as endosymbionts of insects, arthropods, or amoebae [4,7,8]. The scientific and medical interests of characterizing *Chlamydia*-related bacteria are multiple, including the discovery of potential emerging pathogens [9,10,11,12,13] and improved knowledge about infection mechanisms and about the evolution of these strict intracellular bacteria [1,14,15,16,17].

*Estrella lausannensis,* a member of the *Criblamydiaceae* family, was isolated in 2011 from a water sample of the Llobregat river (Spain), [7]. It is an excellent model organism for studying *Chlamydia*-related bacteria as it can replicate in a wide range of cell lines, including human macrophages, Vero cells, insect cells and fish cells, as well as in various amoebae such as *Acanthamoebae castellanii, Dictyostelium discoideum* and *Hartmannela vermiformis* [7,18,19,20,21]. Like other *Chlamydiae*, *E. lausannensis* multiplies using a biphasic life cycle alternating between the infectious form of the bacterium, the elementary body (EB), and the actively dividing form, the reticulate body (RB), which replicates through binary fission [22,23]. A third form of development, the aberrant body (AB), occurs when bacteria are exposed to stress factors such as iron or tryptophan starvation or to growth-inhibiting agents such as interferon-gamma or antibiotics [24]. 

Several studies identified *E. lausannensis* as a possible emerging human pathogen. In particular, a correlation between the presence of anti-*E. lausannensis* antibodies and pneumonia in children was documented (Lienard et al., unpublished data), and it was demonstrated that *E. lausannensis* resist oxidative burst of human macrophages, and even cause a strong cytopathic effect in this cell line [18]. *E. lausannensis* genome sequencing also highlighted important chlamydial virulence factors such as a Type 3 Secretion System (T3SS) and a catalase (KatA) [25,26]. 

In this context, and to determine the pathogenic potential of *E. lausannensis* towards humans, we assessed its ability to grow in the human pneumocyte cell line A549 and evaluated the potential cytopathic effect caused by infection.

## 2. Materials and Methods

### 2.1. Cell Culture and Bacterial Strains

Human pneumocytes A549 (ATCC CCL-185) and HEp2 cells (ATCC CCL-23) were cultivated at 37 °C in the presence of 5% CO_2_ in Dulbecco’s modified minimal essential medium (DMEM, PAN Biotech, Aidenbach, Germany) supplemented with 10% fetal bovine serum (FBS, Gibco, Thermo Fisher Scientific, Waltham, MA, USA). *E. lausannensis* strain CRIB30 [8] was co-cultivated at 32 °C with *Acanthamoeba castellani* strain ATCC 30010 in 25 cm^2^ flasks containing 10 mL of peptone–yeast-extract–glucose broth as described in de Barsy et al. [27].

### 2.2. Infection Procedure

Cells were infected as described in de Barsy et al. [27] with a 1/500 dilution of *E. lausannensis* grown in *A. castellanii*. This dilution represents an MOI of 0.1. 

### 2.3. Quantitative PCR 

Cells were harvested at different time points post infection (0 h, 3 h, 8 h, 24 h, 32 h, 48 h and 72 h post infection) in 1 mL of DMEM. Genomic DNA was extracted from 100 μL following the manufacturer’s instructions (Wizard SV Genomic DNA purification kit, Promega, Madison, WI, USA). qPCR targeting the 16S rRNA gene was performed as described previously by Lienard et al. [8].

### 2.4. Immunofluorescence Staining and Confocal Microscopy

Infected cells were cultivated on glass coverslips. At different time points post infection (0 h, 3 h, 8 h, 24 h, 32 h, 48 h and 72 h), cells were fixed for 10 min in paraformaldehyde 4% at room temperature and washed 3 times with PBS. Immunofluorescence experiments were performed as described in de Barsy et al. [27] with a 1/2000 dilution of a home-made polyclonal rabbit anti-*E. lausannensis* antibody. Secondary antibody (AlexaFluor 488-conjugated donkey anti-rabbit (Life Technologies, Thermo Fisher Scientific, Waltham, MA, USA)) was diluted 1/500 in blocking solution containing 100 ng/mL of Texas Red conjugated-Concanavalin A (Invitrogen, Thermo Fisher Scientific, Waltham, MA, USA) and 150 ng/mL DAPI dilactate (4′,6-Diamidino-2-Phenylindole Dihydrochloride, Molecular Probes, Thermo Fisher Scientific, Waltham, MA, USA). Coverslips were washed twice in PBS + 0.1% saponin, once in PBS and once in deionized water then mounted with 5 μL of Mowiol (Sigma-Aldrich, Buchs, Switzerland). Cells were observed under a confocal microscope (Zeiss LSM 900, Feldbach, Switzerland).

### 2.5. Electron Microscopy

1 × 10^9^ A549 cells were seeded in a 25 cm^2^ flask 24 h before infection, infected as described above with a 1/50 dilution of *E. lausannensis* and harvested 24 h post infection. Cells were fixed and washed as described in de Barsy et al. [27]. Thin sections on grids were prepared by the Electron Microscopy Facility of Geneva University and observed under a Philips EM 201 transmission electron microscope (Philips, Eindhoven, The Netherlands).

## 3. Results

Originally isolated from an amoeba, *E. lausannensis* was previously shown to multiply in a variety of cell lines, including human macrophages [18,20,21,27], and to be associated with pneumonia in children (Lienard et al., unpublished data) To further clarify its potential to cause lower respiratory tract infections in humans, we assessed its ability to grow in the human pneumocyte cell line A549 and evaluated the cytopathic effect of infection.

### 3.1. E. lausannensis Efficiently Replicates in Human Pneumocytes

**Figure 1:**
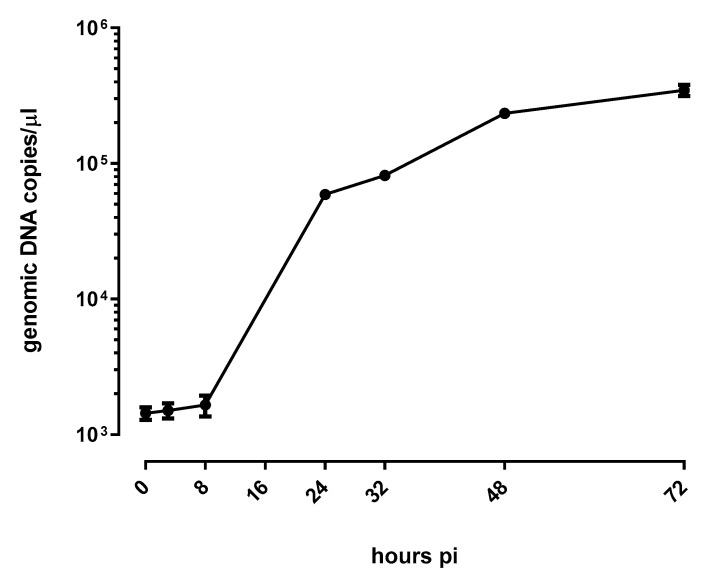
Growth kinetics of *E. lausannensis* in A549 pneumocytes. Bacterial replication is measured using qPCR for 72 h following infection. The results are the mean +**/**− SD of 4 independent experiments.

*E. lausannensis* growth in A549 cells was evaluated using a specific qPCR developed by Lienard et al. [8]. As shown in Figure 1, genomic DNA copies increased by about 2 logs in 72 h, with an exponential replication phase between 8 h and 24 post infection. We observed a lag phase between 0 h and 8 h post infection. During this time, EBs are internalized, differentiate into RBs, and create a replicative niche. After 48 h pi, the number of bacteria reaches a plateau since only a few cells are available for a second round of infection. 

In Table 1, we compare the doubling time of different *Chlamydia*-related bacteria and of *Chlamydia trachomatis* in mammalian (human pneumocytes, human macrophages, Vero cells) and insect cell lines (*S. frugiperda* and *A. albopictus*) as well as in two different free-living protozoa *(A. castellanii* and *D. discoideum*). *E. lausannensis* replication is rapid and efficient in A549 pneumocytes, although slightly slower than in in other mammalian cell lines [18,27]. Its doubling time in pneumocytes is longer than that of *Waddlia chondrophila* [19], but much shorter than that of *Simkania negevensis* [28]. Indeed, *E. lausannensis* shows a longer doubling time than *W. chondrophila* in almost all cell lines tested, except in insect cells, but it grows much faster than *S. negevensis* and *C. trachomatis.*

### 3.2. E. lausannensis Spontaneously Form Aberrant Bodies in Human Pneumocytes and Epithelial Cells

**Figure 2:**
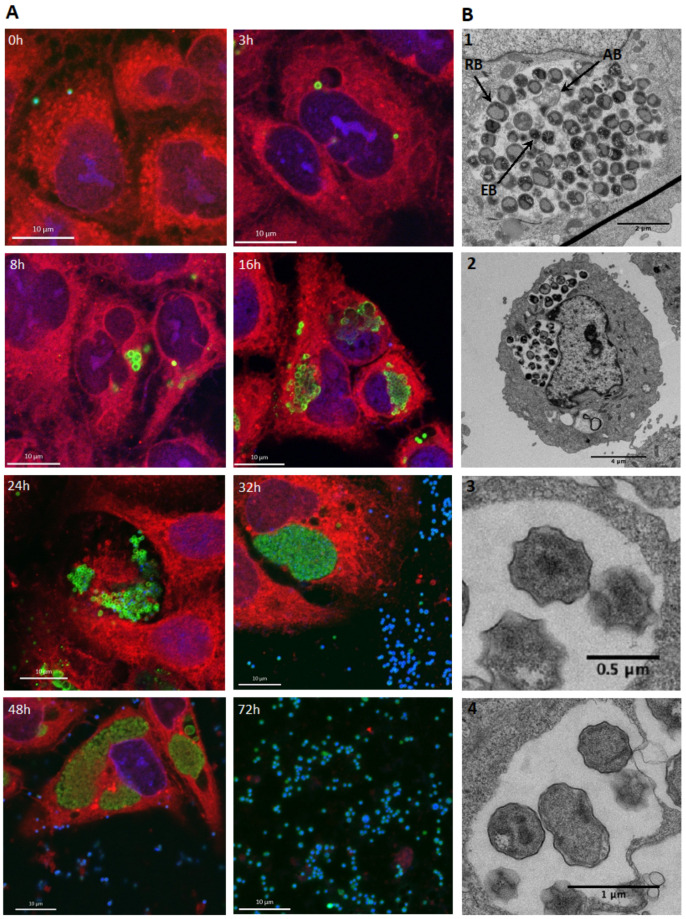
*E. lausannensis* growth in A549. (**A**) Immunofluorescence staining and confocal microscopy at different time points after infection. Bacteria are labelled in green, pneumocytes in red and DNA in blue. (**B**) Electron micrography 24 h post infection showing an inclusion containing EBs, RBs and ABs (1), a pneumocyte with multiple inclusions (2), star-shaped elementary bodies (3) and a dividing RB (4).

We followed the growth of *E. lausannensis* in human pneumocytes using immunofluorescence and confocal microscopy as well as using electron microscopy. As shown in Figure 2A, at 0 h post infection, EBs are localized on the surface of pneumocytes, and at 3 h pi, they start to differentiate into RBs, which are characterized by a larger size and a decondensed chromatin. At 8 h pi, RBs start to replicate, and inclusions with multiple bacteria are visible. At 16 h pi, inclusions are very large and contain multiple bacteria. As of 24 h pi, numerous cells are lysed, and EBs, visualized by their intense DAPI labelling of DNA, are found in the extracellular medium ready to infect new cells. At 72 h pi, all cells are lysed and only extracellular EBs and cellular debris are visible. 

It is interesting to note that in inclusions, DAPI only stained EBsDNA. Decondensed chromatin in RBs is not visible and only late-cycle inclusions when RBs reverted to EBs are clearly labelled with this dye.

Electron microscopy images taken 24 h post infection (Figure 2B) showed the typical star shape of *E. lausannensis* EBs with their condensed chromatin [8]. RBs appear brighter due to chromatin decondensation.

**Figure 3:**
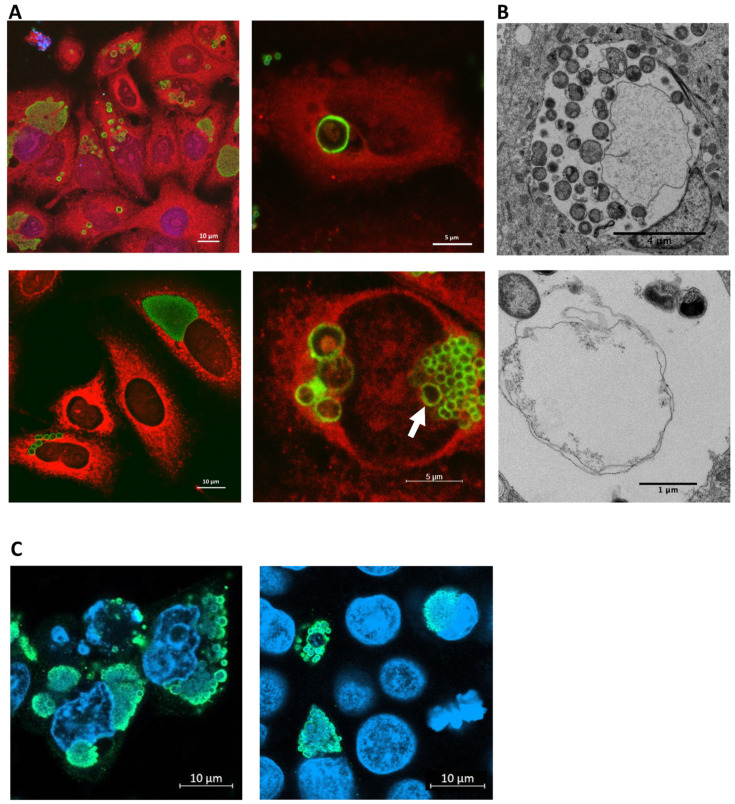
Spontaneous formation of aberrant bodies in human pneumocytes and epithelial cells. (**A**) Immunofluorescence and confocal microscopy 24 h post infection in pneumocytes showing ABs of different sizes (2 to 5 μm) and inclusions containing RBs. White arrow indicates an inclusion containing multiple RBs and one AB. Bacteria are labelled in green, pneumocytes in red and DNA in blue. (**B**) Electron micrography at 24 h post infection showing large ABs in inclusions. (**C**) Spontaneous formation of ABs in HEp-2 cells 16 h pi (image 1) and 24 h pi (image 2).

Interestingly, we observed the spontaneous formation of aberrant bodies (ABs) in some pneumocytes as early as 16 h pi (Figure 3A,B). In these cells, bacteria stopped dividing and their size increased significantly, being around 2 μm when several ABs are present in one inclusion and even exceeding 5 μm when one single aberrant body is present in the inclusion. Such large ABs were also observed using electron microscopy. They appeared as large areas of low electron density, chromatin being possibly localized outside the cut. Interestingly, RBs and ABs were sometimes observed in the same inclusion, although this situation was not predominant.

Using immunofluorescence and confocal microscopy, we could also detect this intriguing spontaneous appearance of aberrant bodies in *E. lausannensis*-infected human epithelial (HEp-2) cells as of 16 h pi (Figure 3C). 

We measured the cytopathic effect of *E. lausannensis* infection in human pneumocytes through propidium iodide incorporation and trypan blue labelling. Both methods revealed a strong cytopathic effect as early as 24 h post infection but failed to quantify host cell mortality at later stages of infection.

## 4. Discussion

In this study, we demonstrated that *E. lausannensis* replicates rapidly in human pneumocytes and that it induces a strong cytopathic effect in this cell line, like what was observed when infecting macrophages or insect cells [18,20]. As observed through confocal microscopy, infected cells undergo strong lysis as of 24 h post infection. This phenomenon of rapid lysis reduces the possible number of replication cycles and limits *E. lausannensis* overall growth (2-log increase in bacterial DNA copy number in A549, compared with 3-log for *W. chondrophila* in the same cell line [19]). In addition, methods such as propidium iodide uptake or trypan blue incorporation, which rely on dye entry into cells with damaged membranes, were unable to quantify dead cells later than 24 h pi. This inability could be explained by the violent cell fragmentation caused by *E. lausannensis* infection and the resulting absence of an intermediate stage between living and completely burst cells. A previous work from Kebbi-Beghdadi et al. demonstrated that *E. lausannensis* replicates in two insect cell lines [20]. In this earlier study, the measurement of propidium iodide incorporation also did not correlate with immunofluorescence observations, suggesting that the same phenomenon of abrupt lysis of host cells occurred in these insect cell lines. 

Our results also highlight the fact that *E. lausannensis* multiplication in mammalian cells, like the multiplication of *W. chondrophila,* is much faster than that of the recognized human pathogen *C. trachomatis*. Both bacteria, again unlike *C. trachomatis,* are also able to grow in a wide variety of host cells, an ability that could be linked to the expression of a large number of OmpA proteins involved in the adhesion to the host cell [25,29]. This hypothesis is reinforced by the observation that another *Chlamydia*-related species, *Parachlamydia acanthamoebae*, which encodes a single Omp protein, multiplies efficiently in only a few cell lines [30].

The most interesting finding of this study is the spontaneous formation of aberrant bodies in human pneumocytes and epithelial cells. Aberrant bodies are defined as enlarged forms of RBs that are persistent and viable, but not proliferative [24]. They appear when bacteria undergo stress such as antibiotics or IFN-gamma treatment [31,32], during nutrient deprivation such as iron deficiency [33,34] or when host cells are co-infected with herpes simplex virus [35]. These forms are reversible, meaning that bacteria resume their replication cycle when the stress stimulus disappears [36]. Spontaneous formation of aberrant bodies is regarded as a form of persistence associated with chronic infection [37]. Aberrant bodies have been observed in vivo in pig enterocytes infected with *Chlamydia suis* [38] and in vitro in human endometrial cells infected with *W. chondrophila* [19]. *E. lausannensis* ABs have also been observed during Vero cell infection in presence of penicillin G but not in untreated cells [27]. 

Scherler et al. demonstrated for *W. chondrophila* that different stress stimuli induce morphologically distinct aberrant bodies [24]. These aberrant bodies are either numerous and small, or very large and few in number It is interesting to note that the morphology of *E. lausannensis* aberrant bodies observed in human pneumocytes and epithelial cells is heterogeneous, ranging from numerous small ABs to a single very large one, and that both types can be present in the same cell. When observed using electron microscopy, *E. lausannensis* ABs appear clearer than EBs and RBs, similarly to what was observed with ABs of *Chlamydia suis* [38]. Furthermore, *E. lausannensis* infection in pneumocytes induces a very strong cytopathic effect, despite the presence of ABs, contrarily to what happens with *W. chondrophila,* where infection in human endometrial cells causes a lesser cytopathic effect than in cell lines in which this species does not form ABs [19]. Because of this cytopathic effect, the evolution of *E. lausannensis* ABs is difficult to study in A549 cells, and it is not possible to determine whether ABs have the potential to resume their cycle and in which conditions this could occur. 

If *E. lausannensis* ABs spontaneously appear in the context of a natural infection, they could contribute to pathogenicity by reducing bacterial visibility by the immune system [37]. They could also provoke a chronic asymptomatic infection, as is the case in 70–90% of *C. trachomatis* infections [39], or cause a latent infection capable of reactivating under certain conditions, such as co-infection with another microorganism, similar to what was described in pigs infected with *C. suis* and *Salmonella* sp [38]. Given the rapid lysis of infected pneumocytes by *E. lausannensis*, this aberrant form could also be a bacterial mechanism to avoid a rapid and detrimental destruction of host cells. 

To conclude, this study supports the hypothesis that *E. lausannensis* is an emerging pathogen potentially able to cause lower respiratory tract infections in humans. The spontaneous formation of aberrant bodies in human pneumocytes and epithelial cells is intriguing and should be further studied. It would be particularly interesting to know if spontaneous aberrant bodies formation is restricted to human cells or if it can also be observed in other mammalian cell lines. Ultimately, the presence of these aberrant forms should also be investigated in vivo in an animal model of *E. lausannensis* infection.

## Figures and Tables

**Table 1:** Doubling times (in hours) of *Estrella lausannensis*, *Simkania negevensis*, *Waddlia chondrophila* and *Chlamydia trachomatis* in different cell lines. Nd = not determined. For *E. lausannensis* in A549, the doubling time was calculated with Doubling Time Calculator using values of 8 h and 24 h post infection.

	*Estrella*	*Simkania*	*Waddlia*	*Chlamydia*
	*lausannensis*	*negevensis*	*chondrophila*	*trachomatis*
	[20] and this work	[28]	[20]	[28]
				
*H. sapiens* pneumocytes	**3.94**	12.89	1.9	nd
*H. sapiens* macrophages	2.75	nd	2.71	nd
*C. aethiops* epithelial	2.53	nd	1.19	11.42
*S. frugiperda*	3.39	21.07	3.78	nd
*A. albopictus*	3.5	nd	4.31	nd
*A. castellanii*	2.71	16.38	1.95	nd
*D. discoideum*	14.45	nd	10.91	nd

## Data Availability

The datasets generated and/or analyzed during the current study are available from the corresponding author on reasonable request.
